# An anomaly in quantum phases induced by borders

**DOI:** 10.1038/s41598-020-63555-x

**Published:** 2020-04-24

**Authors:** Jun Jing, Mike Guidry, Lian-Ao Wu

**Affiliations:** 1 Zhejiang Province Key Laboratory of Quantum Technology and Device, Department of Physics, Zhejiang University, Hangzhou, 310027 Zhejiang China; 2 Department of Theoretical Physics and History of Science, The University of the Basque Country (EHU/UPV), PO Box 644, 48080 Bilbao, Spain; 3 Department of Physics and Astronomy, University of Tennessee, Knoxville, Tennessee 37996 USA; 4 Ikerbasque, Basque Foundation for Science, 48011 Bilbao, Spain

**Keywords:** Physics, Quantum physics, Quantum information, Quantum mechanics

## Abstract

The stationary behavior of a quantum system is determined by its Hamiltonian and its boundary conditions. All quantum phase transitions (QPT) reported previously were induced by changing the Hamiltonian. In a circular spin model with Heisenberg XY interactions and no magnetic field, we observe an anomaly in quantum phases caused by a qualitative change of the boundary condition. The unexpected anomaly features an infinite number of single-particle levels, in the same pattern as the single-photon-triggered quantum phase transition in the Rabi model.

## Introduction

Phase transitions are common macroscopic/bulk phenomena in both classical and quantum mechanical regimes^1–4^. They can be organized into three basic categories: (i) discontinuous or first-order phase transitions^5^, (ii) continuous or second-order phase transitions^6^, and (iii) topological Kosterlitz - Thouless transitions between bound vortex-antivortex pairs at low temperatures and unpaired vortices and anti-vortices at high temperatures^7–10^. The ground state of a quantum system is the resource that enables quantum adiabatic passage^11–13^ and quantum annealing^14–16^, which are of great relevance to quantum information theory, quantum computation^17^, and quantum control^18^. It is then important to understand the conditions under which a system can experience a quantum phase transition that alters fundamentally the structure of the ground state. The quantum structure for the wavefunction is in general a more sensitive probe than the energy of the ground state. It is expected that the qualitative changes of the ground-state wavefunction at phase transitions could have a dramatic effect on its role in quantum information processing.

The basic features of quantum phase transitions have been reasonably well understood^19–21^. As summarized by Sachdev^1^, a first-order quantum phase transition implies a sudden change of the ground-state wavefunction, accompanied by a singularity in the ground-state energy and a level-crossing or an avoided crossing between the two lowest states. A second-order or continuous quantum phase transition exhibits a singularity in the derivatives (in any order) of the ground-state energy with respect to some order parameter at the critical point, which is associated with the scaling behavior such as bipartite entanglement of a many-body system^19,22,23^. While quantum phase transitions often occur in *many-particle* systems, recently *single-particle* (photon)-triggered quantum phase transitions have been found in the single-boson Rabi model^20,21^.

A typical quantum phase transition is tuned by varying a parameter that controls the strength of a perturbation: for example, an external transverse magnetic field that disrupts the magnetic order of a quantum Ising model^19,21,24,25^, applied pressure, or the level of doping with electron donors or acceptors. However, the dynamics of a quantum system is governed not only by its Hamiltonian but also by its boundary conditions. Therefore we ask: can an anomaly in quantum phases similar to a quantum phase transition be induced by boundary conditions? Study of such effects could be fertile ground for discovering new classes of anomalies in quantum phases or quantum phase transitions that might enrich our perspectives in understanding quantum mechanics. This work reports new anomalies in quantum phases of single-particle systems driven by tuning of boundary conditions. Similar to the single-photon quantum phase transition in the Rabi model^20,21^, the anomaly occurs in a system consisting of finite number of excitons or particles but with an infinite number of sites or within an infinite Hilbert space. Thus our model is another manifestation of a phase transition that extends the conventional concept of quantum phase transitions in *many-particle* systems to systems that are *single-particle* in nature but with an *infinite number of degrees of freedom*.

**Figure 1:**
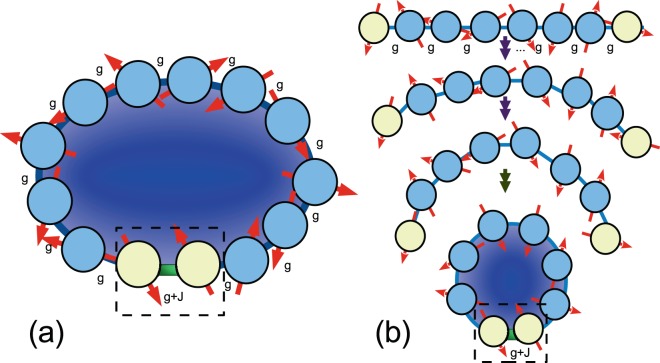
A model system that demonstrates an anomaly in quantum phases. (**a**) This one-dimensional spin system with a finite number of sites *N* can be viewed as the sum of two topologies: a spin-circle with isotropic interaction among nearest-neighbor sites and a spin-line segment consisted of two sites with arbitrary coupling strength. (**b**) With increased or decreased bending, the coupling between the two ends of the system becomes stronger or weaker.

In particular, the hidden anomaly in the quantum phase is found to be induced by tuning the microscopic coupling strength *J* between a single pair of nearest-neighbor sites along a closed one-dimensional spin system. This one-dimensional model of Heisenberg XY exchange interaction is demonstrated in Fig. 1. It can be obtained from the hard-core Boson Hubbard model having *N* sites with a single hard-core boson, i.e., the average particle occupation for each site is *n*_0_ = 1/*N*. Thus in the thermodynamical limit *N* → ∞, the system approaches a Mott insulator with *n*_0_ = 0. A complete analysis may be found in Method.

The Hamiltonian of the total system is written as

1 $$\begin{array}{rcl}{H}_{{\rm{tot}}} & = & {H}_{0}+H(J),\\ {H}_{0} & = & \frac{g}{2}\mathop{\sum }\limits_{n=1}^{N}({X}_{n}{X}_{n+1}+{Y}_{n}{Y}_{n+1}),\\ H(J) & = & \frac{J}{2}({X}_{s}{X}_{s+1}+{Y}_{s}{Y}_{s+1}),\end{array}$$

where *g* is the exchange coupling strength between nearest neighbor spins, and *X* and *Y* indicate the Pauli matrices *σ*^*x*^ and *σ*^*y*^, respectively. The index *s* is an arbitrary site number ranging from 1 to *N*. Units will be so chosen that the coupling strength *g* is equal to one in the following discussion. The full exciton number is conserved because $[{H}_{{\rm{tot}}},\,{\sum }_{n}{Z}_{n}]=0$, where ${Z}_{n}\equiv {\sigma }_{n}^{z}$. The quantum phase transition of interest can be observed in the subspace with one exciton. Note that under such conditions the constituents at sites of the system can be replaced by harmonic oscillators (modelling coupled single-mode cavities) or other bosonic modes. Hence the interaction term for two sites can be expressed as ${a}_{n}^{\dagger }{a}_{n+1}+{a}_{n+1}^{\dagger }{a}_{n}$, where *a* (*a*^†^) is the annihilation (creation) operator. Our Hamiltonian (1) is reminiscent of many models (for example, ref. ^26^) that are used to study impurity or disorder effects in the one-dimensional XY model or Heisenberg model. In contrast to these works, the existence of an impurity in the bulk will destroy the *U*(1) and *Z*_2_ symmetries in our Hamiltonian and the critical behavior observed here (the dramatic distinction between the ground states in the single-exciton subspace with *J* < 0 and *J* ≥ 0) has not been reported previously.

## Results

### Symmetry analysis of the system

In the language of network topology the model of Eq. (1) describes a hybrid system consisting of a circle or ring topology and a line segment or bus topology. Figure 1(a) illustrates that the connecting strengths between the nearest-neighbor sites are homogenous except that between the pair *s* and *s* + 1, which is equivalent to the addition of a small line segment described by *H*(*J*). In the following analysis, we choose *s* = *N* (then *s* + 1 = 1) with no loss of generality, i.e., we concentrate on the boundary condition between the neighbor sites 1 and *N* in periodic condition. When *J* = −1 (in units where *g* = 1), the whole system is an open-ended line segment. When *J* = 0, the system becomes a circle that is translational-invariant along the sites. The line and the circle are not homeomorphic because the line can be disconnected by removing one site but the circle can not. The circle and the line however are locally homeomorphic only when they have an infinite number of sites. When *J* > 0 and −1 < *J* < 0 the system has no translational invariance. Thus our anomaly in quantum phases is induced by strengthening the boundary sites in the close-ended condition. In contrast, in the single-photon-triggered Rabi model^20,21^, the phase transition occurs in the limit that the energy splitting of the qubit is much larger than that of the bosonic mode. Thus in the strong-coupling regime the population of the bosonic mode in the ground state can be enhanced suddenly from zero when the coupling strength reaches the critical value.

For the physical realization of our model, as *J* is increased from −1 to positive values the system can be regarded as a spin-line segment with increased bending [see Fig. 1(b)]. This effectively changes the boundary condition of the system because of the increasing coupling between the spins at the two ends. This behavior may be expected to occur for any physical system where the site-site interaction is proportional to certain powers of the site separation distance. For example, a Coulomb-like interaction is inversely proportional to the square of the distance between the subsystems and a 10% variation in the site-site distance yields a greater than 25% variation in the mutual coupling strength. Then when the one-dimensional system is folded as in Fig. 1(b) until its two ends approach each other, their mutual interaction depends sensitively on their separation distance. Thus the model in Eq. (1) can also be used to investigate the effect of boundary conditions. In a large system with *N* sites the boundary terms are conventionally expected to be of order 1/*N*, and thus to have negligible influence on macroscopic physical quantities. However, we find in this model that microscopic boundary conditions can have a dramatic effect because they can lead to an anomaly in quantum phases.

Henceforth we confine attention to the effect of the control parameter *J* in Eq. (1). Roughly the passage from *J* = −1 to *J* = 0 is expected to be connected closely to the transition from one well-defined topology to another well-defined topology, while the passage from *J* = 0 to *J* > 0 corresponds to a transition from a well-defined topology to a hybrid one. As will now be demonstrated, we observe an anomaly in quantum phases around *J* = 0 in this system that is reflected in the singularity properties of the ground and the first-excited states.

### Ground state properties

**Figure 2:**
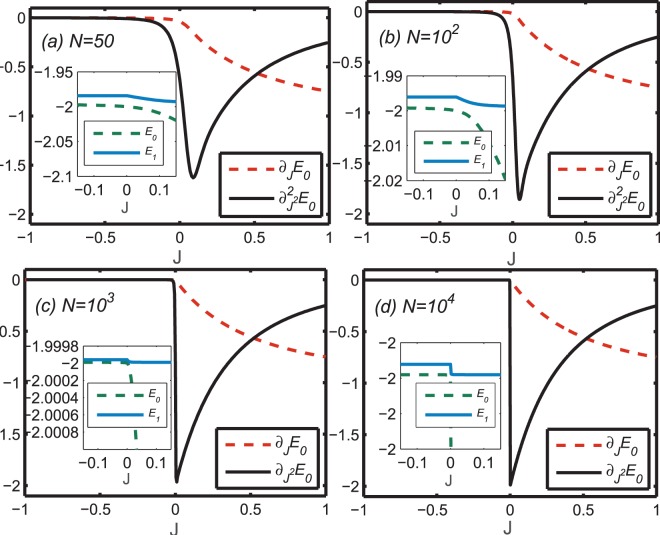
The first derivative of the ground state energy with respect to *J* (dashed red curve) and the second derivative (solid black curve) as functions of *J*. The sizes of the system are (**a**) *N* = 50, (**b**) *N* = 10^2^, (**c**) *N* = 10^3^, (**d**) *N* = 10^4^, respectively.

To understand the singularity in the ground state, we first consider the dependence of the ground state energy on system size, which is a functional of the boundary condition parameterized by *J*, or the extra strength between two selected neighbor sites 1 and *N*. We display the two lowest derivatives of the ground state energy *E*_0_ with respect to *J* ∈ [−1,1] in Fig. 2(a–d) for systems with *N* = 50, 100, 1000, and 10,000 sites, respectively. The variation of the energies for the ground and the first excited states as functions of *J* indicates an anomalous transition from a gapless phase to a gapped phase. Neither derivative changes significantly until *J* approaches zero. The first derivative ${\partial }_{J}{E}_{0}$ exhibits a rapid decrease with increasing *J* after passing through *J* = 0, while the second derivative ${\partial }_{{J}^{2}}^{2}{E}_{0}$ exhibits a cusp at *J* = 0, with both behaviors becoming more sharply defined as the site number is increased. Thus, the variation of the energy derivatives indicates that the model experiences an anomaly at the critical point *J* = 0 that is controlled entirely by tuning the connection strength between the spin pair on the boundary.

**Figure 3:**
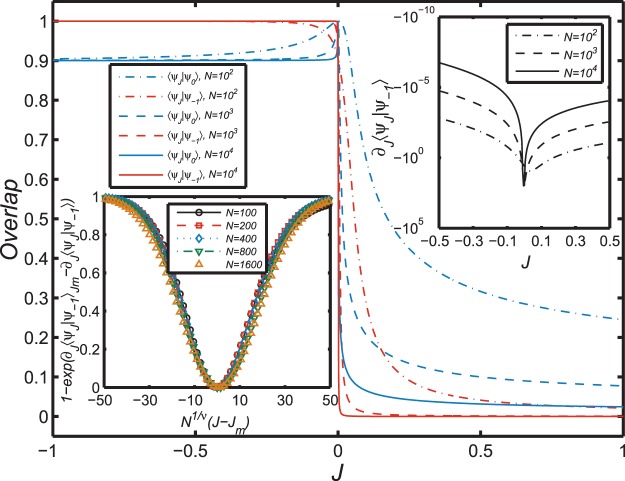
The overlap between the ground state $|{\psi }_{J}\rangle$ within the interval *J* ∈ [−1, 1] and the special case with $|{\psi }_{-1}\rangle \equiv |{\psi }_{J=-1}\rangle$ (red lines) and that with $|{\psi }_{0}\rangle \equiv |{\psi }_{J=0}\rangle$ (blue lines). Different sizes of the system are represented by different type of lines (dot-dashed lines for *N* = 10^2^, dashed lines for *N* = 10^3^, and solid lines for *N* = 10^4^). Top-right inset: The first derivative of the overlap $\langle {\psi }_{J}|{\psi }_{-1}\rangle$ with respect to *J* for different system sizes. Lower-left inset: Finite-size scaling of the derivative ${\partial }_{J}\langle {\psi }_{J}|{\psi }_{-1}\rangle$ with the number of sites *N*. The derivative ${\partial }_{J}\langle {\psi }_{J}|{\psi }_{-1}\rangle$ is a function of *N*^1/*v*^(*J* − *J*_*m*_) with a critical exponent *v* ≈ 0.63, and *J*_*m*_ is the position of the minimum of $\langle {\psi }_{J}|{\psi }_{-1}\rangle$. All the data from *N* = 100 to *N* = 1600 collapse onto a single curve.

A more sensitive indicator of the anomaly is afforded by changes of the wavefunction near the critical point. Figure 3 displays the overlap of the ground state wavefunction $|{\psi }_{J}\rangle$ with the wavefunctions $|{\psi }_{-1}\rangle \equiv |{\psi }_{J=-1}\rangle$ and $|{\psi }_{0}\rangle \equiv |{\psi }_{J\mathrm{=0}}\rangle$. The ground state wavefunction overlaps exactly the initial state $|{\psi }_{-1}\rangle$ as *J* is increased along the interval *J* ∈ [−1,0], and then transforms rapidly into $|{\psi }_{0}\rangle$ near the single point *J* = 0, with the transition becoming increasingly sharp with a larger *N*. When *N* is increased to 10,000, the overlap exhibits a critical behavior through its dependence upon *J*, the corresponding transition becomes essentially a step function, and the derivative ${\partial }_{J}\langle {\psi }_{J}|{\psi }_{-1}\rangle$ shown in the top-right inset in Fig. 3 forms an increasingly clear cusp as *N* increases. In the limit *N* *→* ∞, the quantity ${\partial }_{J}\langle {\psi }_{J}|{\psi }_{-1}\rangle {|}_{J{\mathrm{=0}}^{+}}$ is logarithmically divergent^19,22^. This result is seen here to be a direct consequence of the singular behavior of ${\partial }_{J}\langle {\psi }_{J}|{\psi }_{-1}\rangle$, which exhibits the finite-size scaling shown in the lower-left inset of Fig. 3. The critical exponent *v* is found numerically to be around 0.63. Even more interesting, after *J* increases through the critical point *J* = 0 the ground state wavefunction jumps immediately to another state $|{\psi }_{J\mathrm{ > 0}}\rangle$ that is fully orthogonal to $|{\psi }_{-1}\rangle$ (their overlap vanishes) and nearly orthogonal to $|{\psi }_{J\mathrm{=0}}\rangle$ (their overlap is also very close to zero). Thus Fig. 3 indicates unequivocally an anomalous transition at *J* = 0 involving a level crossing in which the ground state is replaced by a new ground state essentially orthogonal to the original state. Combining these results with those for the derivatives of the ground state energy in Fig. 2, this new kind of anomaly can be categorized as a single-exciton quantum phase transition that is of second order.

## Discussion

The preceding results are remarkable in that the anomaly in quantum phases may be attributed entirely to a microscopic boundary condition of the quantum system, without invoking any internal or applied external fields. According to the Hellmann - Feynman theorem^27^,

2 $${\partial }_{J}{E}_{0}=\langle {\psi }_{J}|{\partial }_{J}{H}_{{\rm{tot}}}|{\psi }_{J}\rangle =\frac{1}{2}\langle {\psi }_{J}|({X}_{1}{X}_{N}+{Y}_{1}{Y}_{N})|{\psi }_{J}\rangle ,$$

where $|{\psi }_{J}\rangle$ denotes the ground wavefunction of the system with special *J*. Thus the expectation value of the boundary interaction term *X*_1_*X*_*N*_ + *Y*_1_*Y*_*N*_ may be taken as the order parameter, in analogy with the single-photon-triggered quantum phase transition in the Rabi model. The dimensionless interaction strength *J* between a single pair of sites along the circle, which is a quantity that could be modified easily in experiments, previously was thought to be unimportant in the thermodynamical limit *N* → ∞.

Therefore one can see that the transition of the system from a chain to a circle as *J* varies from *J* = 1 (a mixture of line-segment and circle topologies) to *J* = 0 (a perfect circle) *causes no phase transition* due to the invariance of the ground state. However, the transition of the *boundary condition* from a state with translation symmetry (*J* = 0) to a state of broken symmetry (*J* > 0) leads to a clear transition due to a two-step sudden change in the ground state. After the critical-point, the ground state becomes ${\psi }_{-1}^{\perp }$ for *J* > 0 up to a global phase, satisfying $\langle {\psi }_{-1}^{\perp }|{\psi }_{-1}\rangle =0$.

The two quantum phases separated by the critical point *J* = 0 are characterized by dramatic differences in the properties of the ground state when −1 < *J* ≤ 0 and when *J* > 0. In the limit of large *N*, the ground state for −1 < *J* ≤ 0 is expected to be

3 $$|{\psi }_{-1}\rangle =\sqrt{\frac{2}{N+1}}\mathop{\sum }\limits_{k=1}^{N}\,\sin \left(\frac{kN\pi }{N+1}\right){\sigma }_{k}^{+}\mathrm{|0}\rangle \mathrm{}.$$

**Figure 4:**
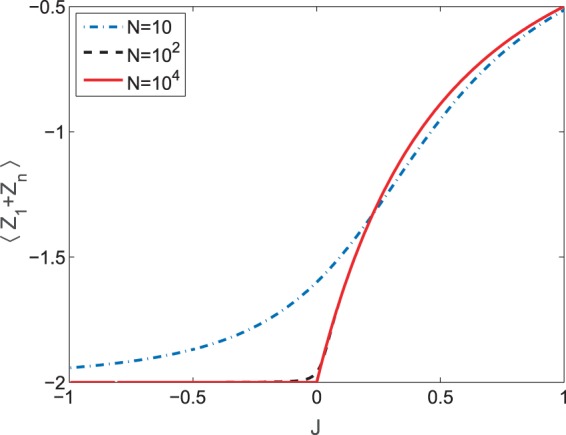
The expectation value of *Z*_1_ + *Z*_*N*_ over the ground state is plotted for different sizes of system to indicate the population distribution over the spin-flip in the two ends. It is also used to imply the localization property of the ground state.

However, this result will fail as *J* → 0^+^ from *J* ≤ 0, as indicated by Fig. 2. In the limit *N* → ∞, the ground state becomes the ground state of ${X}_{N}{X}_{1}+{Y}_{N}{Y}_{1}$, which is ${\mathrm{(|0}\rangle }_{N}\mathrm{|1}{\rangle }_{1}-\mathrm{|1}{\rangle }_{N}\mathrm{|0}{\rangle }_{1})/\sqrt{2}\otimes {\prod }_{k\ne \mathrm{1,}N}\mathrm{|0}{\rangle }_{k}$. The population distributions over the bases of these two ground states with respect to $J$ are obviously different, as shown in Fig. 4. For large *N* one finds that ${\langle {Z}_{1}+{Z}_{N}\rangle }_{{\psi }_{J}}=-\,2$ for −1 < *J* ≤ 0, which means that population of the spin-flip on the boundary (the two ends) is negligible in this conventional range. In contrast, ${\langle {Z}_{1}+{Z}_{N}\rangle }_{{\psi }_{J}}$ increases suddenly at the critical point *J* = 0^+^, and this effect on the boundary becomes sharper as the size of system increases. Thus the system exhibits a clear transition from a delocalized phase to a localized one at the critical point, analogous to that found in the Rabi model^28^.

**Figure 5:**
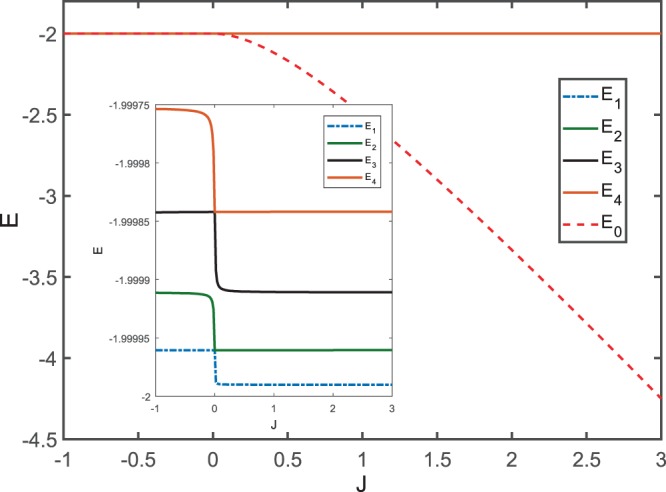
Eigenvalues of the lowest 5 eigenstates as a function of *J*. Inset: the first 4 quasi-degenerate excited states. The size of the system is chosen as *N* = 10^3^.

Our work also shows that the anomaly in quantum phase can be viewed as a transition between a gapless phase and a gapped one. Figure (5) provides a detailed plot of the eigen-energy structure for our model with *N* = 10^3^. One can clearly see that the energies of the states labelled *E*_1_ to *E*_4_ are quasi-degenerate for all *J*, while the ground-state energy *E*_0_ (see the dashed red line) drops suddenly just when *J* moves over 0^+^ along the direction from the negative to the positive axis.

The microscopic details of the individual building blocks of a system generally are believed to not be important to quantum phase transitions. Rather, the collective behavior is thought to be controlled by general properties of the interaction between the building blocks (spins or harmonic oscillators), and the quantum phase transition is induced by tuning an “external” parameter (field strength, pressure, doping concentration, …). We have exhibited here a new class of anomalies in quantum phases induced by a microscopic boundary condition as well as translational symmetry in a finite-size, one-dimensional spin model with no external field applied that defies this common understanding. The anomaly in quantum phases exhibited here does not require a noncommuting condition between different constituents of the Hamiltonian. At the critical point, we demonstrate a nonanalytic behavior of the second-order derivative of the ground eigen-energy and a sudden change of the ground state wavefunction. This special anomaly in quantum phases might also be understood using concepts developed for symmetry-protected topological phases except that the system analyzed here has a *local order parameter*. With respect to the analytical result, we apply the Jordan-Wigner transformation to get the ground state (3) when the additional boundary coupling strength *J* ≤ 0, while have to resort to the numerical simulation when *J* moves over the critical point 0^+^ to the other phase regime. It is then interesting to explore the effects of boundary condition in other strongly correlated systems in the future with the numerical methods, such as the Monte Carlo simulation^29^ and the dynamical mean-field theory^30^.

## Method

Optical lattices of ultracold atoms offer a realization of the boson Hubbard model, and exhibit the superfluid - insulator transition^31^. Here we shall present a hidden anomaly in quantum phases in a model that could be realized by a relevant physical system described by a one-dimensional boson Hubbard model; it is of interest for quantum computation, quantum information, and ultra-cold atoms^4,32^. The Hamiltonian of the boson Hubbard model neglecting off-site and longer-range repulsions consists of three terms,

4 $$H=-\,g\sum _{n}({b}_{n}^{\dagger }{b}_{n+1}+h\mathrm{}.c\mathrm{.)}-\mu \sum _{n}{b}_{n}^{\dagger }{b}_{n}+\frac{U}{2}({b}_{n}^{\dagger }{b}_{n}-\mathrm{1)}{b}_{n}^{\dagger }{b}_{n}\mathrm{}.$$

Here the first term allows site-hopping of the bosons through the creation and and annihilation operators ${b}_{n}^{\dagger }$ and *b*_*n*_, with *g* the hopping matrix element, *μ* in the second term represents the chemical potential of bosons, and the on-site repulsion *U* in the third term sets the energy scale for the problem. By relying on specific physical conditions, a given system can be constrained to have a fixed total number of bosons. We study in this work a hard-core boson Hubbard model having *N* sites, with a single hard-core boson that can also be represented as a spin-$\frac{1}{2}$ particle. In the thermodynamical limit *N* → ∞ the system approaches a Mott insulator^33^. For an open-ended chain or a closed homogeneous ring, the Heisenberg XY model can be solved analytically using a Jordan - Wigner transformation^34–36^, which converts a spin-$\frac{1}{2}$ system into a free spinless fermion chain with nearest-neighbor hopping.

For our model, in the limit of hard-core bosons on every site of the lattice in a Hubbard model, only the two Mott insulator states with *n*_0_ = 0 or *n*_0_ = 1 are permitted. This model can be mapped to a magnet of *S* = 1/2 spins with nearest-neighbor exchange interactions. The Hamiltonian is written as

5 $${H}_{{\rm{X}}X}=-\,\frac{g}{2}\sum _{\langle ij\rangle }({\sigma }_{i}^{x}{\sigma }_{j}^{x}+{\sigma }_{i}^{y}{\sigma }_{j}^{y})+\frac{\mu }{2}\sum _{i}{\sigma }_{i}^{z}=-\,g\sum _{\langle ij\rangle }({\sigma }_{i}^{+}{\sigma }_{j}^{-}+{\sigma }_{i}^{-}{\sigma }_{j}^{+})+\frac{\mu }{2}\sum _{i}{\sigma }_{i}^{z}.$$

Under the *periodic boundary condition*, the Hamiltonian (5) for the one-dimensional case can be rewritten as

6 $${H}_{{\rm{X}}X}^{CB}=-\,g\mathop{\sum }\limits_{i\mathrm{=1}}^{N}({\sigma }_{i}^{+}{\sigma }_{i+1}^{-}+{\sigma }_{i}^{-}{\sigma }_{i+1}^{+}+w{\sigma }_{i}^{z}),$$

where $w\equiv -\,\mu \mathrm{/(2}g)$. Now we perform the Jordan-Wigner transformation by

7 $${c}_{i}=(\prod _{1\le j < i}{\sigma }_{j}^{z}){\sigma }_{i}^{-}=(\prod _{1\le j < i}{e}^{i\pi {\sigma }_{j}^{-}{\sigma }_{j}^{+}}){\sigma }_{i}^{-}={e}^{i\pi {\hat{n}}_{i\downarrow }}{\sigma }_{i}^{-},$$

where ${\hat{n}}_{i\downarrow }\equiv {\sum }_{1\le j < i}{\sigma }_{j}^{-}{\sigma }_{j}^{+}$ is the number operator counting the number of the holes from site-1 to site-(*i* − 1). Up to *i* = *N* + 1, we have ${c}_{1}={\sigma }_{1}^{-}$ and ${c}_{N+1}={e}^{i\pi {\hat{n}}_{\downarrow }}{\sigma }_{N+1}^{-}={e}^{i\pi {\hat{n}}_{\downarrow }}{\sigma }_{1}^{-}$, where ${\hat{n}}_{\downarrow }\equiv {\hat{n}}_{N+1\downarrow }$. Note ${c}_{N+1}\ne {c}_{1}$ although ${\sigma }_{N+1}^{-}={\sigma }_{1}^{-}$. It gives rise to

8 $${H}_{{\rm{X}}X}^{CB}=-\,g[w\mathop{\sum }\limits_{i=1}^{N}\mathrm{(1}-2{c}_{i}{c}_{i}^{\dagger })+\mathop{\sum }\limits_{i=1}^{N-1}({c}_{i}{c}_{i+1}^{\dagger }+{c}_{i+1}{c}_{i}^{\dagger })+{e}^{i\pi ({\hat{n}}_{\downarrow }+\mathrm{1)}}({c}_{N}{c}_{1}^{\dagger }+{c}_{1}{c}_{N}^{\dagger })]\mathrm{}.$$

The boundary term breaks the periodicity of the Jordan-Wigner operators. Then we can define a deformed Fourier transition

9 $${c}_{j}=\frac{1}{\sqrt{N}}{e}^{\frac{2\pi i\alpha j}{N}}\mathop{\sum }\limits_{k=1}^{N}{e}^{\frac{2\pi ikj}{N}}{b}_{k},$$

where *α* = 0 mod *N* if *n*_↓_ is odd and $\alpha =\frac{1}{2}$ mod *N* if *n*_↓_ is even. Up to a constant number, we have

10 $${H}_{{\rm{X}}X}^{CB}=-\,2g\mathop{\sum }\limits_{k=1}^{N}\left[w-\,\cos \left(2\pi \frac{\alpha +k}{N}\right)\right]{b}_{k}^{\dagger }{b}_{k}\mathrm{}.$$

With one exciton/fermion/spin-up/particle and setting *g* = 1, the energy density (off the vacuum energy) is

11 $${\varepsilon }_{k}^{\mathrm{(1)}}=-\,\frac{2}{N}\left[w-\,\cos \left(2\pi \frac{\alpha +k}{N}\right)\right]\mathrm{}.$$

Thus, in the one-exciton subspace we can easily obtain *k* = *N*/2 that minimizes the energy for an even *N* (in the *α* = 0 sector) and *k* = (*N*– 1)/2 that minimizes the energy for odd *N* (in the $\alpha =\frac{1}{2}$ sector). In this case, the ground state energy (up to a c-number) is −2 when *g* = 0. Suppose *N* is even, then *α* = 0 and the ground state reads $|{\psi }_{gs}^{\mathrm{(1)}}\rangle ={b}_{N\mathrm{/2}}^{\dagger }\mathrm{|0}\rangle$. That yields Eq. (3) in the main text.
